# Learn to Walk

**Published:** 1892-04

**Authors:** 


### LEARN TO WALK.

Americans are, for the most part, bad walkers. It is rare to find an exception, even in our army. Among Europeans, and the aborigines of our own continent, a noble mien is not uncommon. I understand the causes of this ugly defect among our people, and my present purpose is to call attention to it, and to point out the remedy.

In English and French books on the military drill and physical training whole chapters discuss the subject of walking. We are told that this or that part of the foot must touch the ground first, that the angles must be so and so, etc. I will not say this advice is not right, but I will say that very few have been helped by it.

Look at a good walker. Shoulders, head and hips drawn well back and the chest thrown forward. What a firm, vigorous tread! Such a walk may easily be secured by carrying a weight upon the head. An iron crown has been devised for this purpose. It consists of three crowns one within the other, each weighing about nine pounds. One or all three may be worn at a time.

The water-carriers of Southern Europe, although belonging to the lowest class, have a noble bearing. Certain negroes in the South, who "tote" burdens upon the head as a business, can readily be pointed out in a crowd. The effort required to keep the burden directly over the spine so develops the muscles of the back and neck that in the absence of the burden, the head is carried in a noble, erect attitude.

By carrying one of these crowns upon the head half an hour two or three times a day while walking in the garden or through the halls of the house, one may soon become a fine walker. One-tenth of the time occupied in learning a few tunes on the piano, given to this exercise, would insure any girl a noble carriage. The crown is not necessary. Any weight which does not press upon the very crown of the head, but about it, will answer the purpose equally well.

